# Numerical and Experimental Investigations of the Influence of Operation on the Technical Condition of Pressure Vessels

**DOI:** 10.3390/ma15207281

**Published:** 2022-10-18

**Authors:** Przemyslaw Moczko, Michał Paduchowicz, Damian Pietrusiak

**Affiliations:** Department of Machine Design and Research, Faculty of Mechanical Engineering, Wroclaw University of Science and Technology, 50-370 Wroclaw, Poland

**Keywords:** pressure vessels, fatigue calculations, FEA, residual life estimation

## Abstract

The paper presents the issues related to the design and assessment of the technical condition as well as determination of the residual durability of pressure equipment. Based on a real a example, a liquid nitrogen spherical tank, we present the development and applicability of the method for assessment of the durability of the structure. In terms of the material itself, the authors analyze macroscale (structural) factors of the geometry of the real structure (by 3D scanning: material wear detection, deflections and deformations, etc.) and measured real operational loads to develop an integrated method, including material model and behavior in its operational condition, delivering a useful tool for macroscale structural analyses of the materials under complex load (mechanical, thermal, chemical, etc.). As a result, a detailed analysis of the tank is presented. The paper gives an idea of the method, its development, usefulness, and applicability of the presented approach by indication of the mutual influence of pressure vessel components (e.g., stubs, manholes) and operational loads, which may result in underestimating the strength and durability of the pressure vessels in the design process and during operation.

## 1. Introduction

Chemicals are present in large amounts in industrial installations as a processing medium and as the process output as well. That rises the common safety demand, which may be critical to any industrial installations and the operating team, as well as neighboring habitats. The durability of storage installations may be influenced by the chemicals itself; however, one needs to remember that high pressure levels of the working medium and its cyclical change over time as a result of the course of technological processes, which may be a critical factor for long-lasting safety of the installation. Equipment that belongs to this risk group includes, among others, pressure tanks for liquid nitrogen. This article discusses the issues of forecasting the durability of such a device subjected to many years of operation.

The pressure device design must take into consideration such facts as the necessity of periodic inspections, the periodic/cyclic type of the load and many safety aspects. That brought the necessity to develop design and operation requirements purely dedicated to those kinds of structures. In light of standard documents EN 12953-3 [1] and EN 12953-4 [2], which are commonly used to assess the durability of damage caused by fatigue of pressure equipment, methods of identification of load cycles called “range-pair” are used, and then to count the cycles loads called “rain-flow-load”. In the final stage, for each class of load cycles, unit durability losses are determined, which in the final stage are summed using the load accumulation method, e.g., the Palmgren–Miner linear failure accumulation hypothesis [3,4]. The total loss of durability determined in this way should be less than 1. A similar approach to durability assessment, differing mainly in the method of determination the complex state of stress and deformation in specific areas of the structure (e.g., in the area of welded joints), can be ASME standards: “Code Cases—Boilers and Pressure Vessels [5], TRD 301 [6], or PN-EN 13445-5: 2021-10 [7].

The issues of durability assessment of pressure vessels are also discussed in common available publications. The vast majority of the durability assessment described in them is based on the point values of deformations, stresses derived from the complex state of stress and deformation of the structure, determined using the finite element method [8,9]. On the other hand, further procedures in the durability assessment presented in the exemplary publications [10] or [11] are analogous to those in standard documents. Another way to approach the assessment of durability is using the so-called criteria functions, such as the parameters of Coffin and Ostergren, which have been described in publications prepared for NASA [12,13]. Articles [14,15] refer to the durability of the super-heater chamber, which was determined on the basis of the critical plane defined with the use of two-directional angles. For the hysteresis loop corresponding to this plane, the Ostergren parameter was determined, which was then referred to the actual criterion function describing the dependence of the Ostergren parameter on the number of cycles to failure determined on the basis of tests on samples.

Moreover, for the evaluation of the durability of devices operating under thermo-mechanical fatigue conditions, a new parameter called P was proposed in the publication [16]. This parameter takes into account the influence of the stress amplitude, the range of plastic deformation and temperature.

Nevertheless, most of the published materials, including those mentioned above, deal with material issues in microscale or local behavior of cylindrical components. In the presented article, the authors target the gap between large amounts of research describing materials and structures in local (micro/mesoscale) and the practical application of the knowledge in the structural integrity of the structures (macroscale). None of the presented publications brings the applicability of the knowledge as a method useful for the engineering staff facing structural long-life issues in real operation. We find it very common that very detailed microscale models are not applicable in the case when real size structure must be analyzed. In this paper, we present a practical method that uses the history of the equipment operation, basic mechanical and strength properties of construction materials, and numerical methods in forecasting further available durability of pressure equipment, including liquid nitrogen tanks, with respect to the development of knowledge in computing, measurements and material sciences.

## 2. Materials and Methods

**Figure 1:**
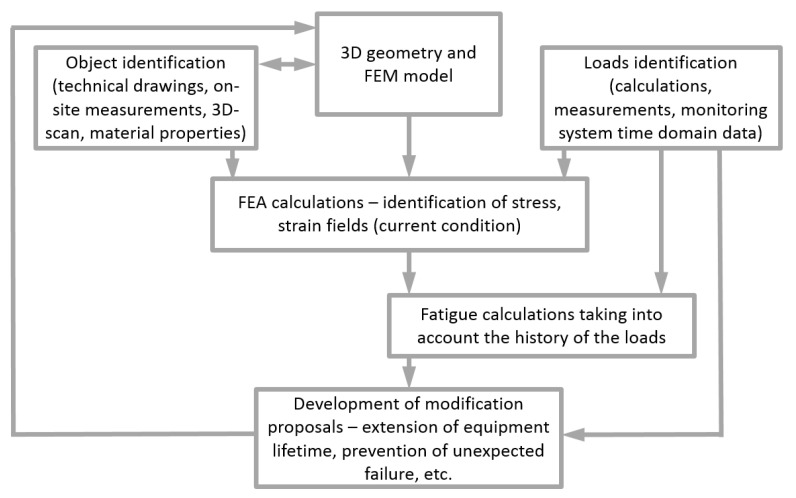
Method for assessing the condition of pressure equipment.

The proposed method for assessing the technical condition and durability of pressure vessels after their long-term operation is based on the results of advanced simulation analyses and experimental tests, experimental data in the field of the current technical condition, history of operating loads, and other information relevant to the assessment. A diagram of the proposed method is shown in Figure 1.

**Figure 2:**
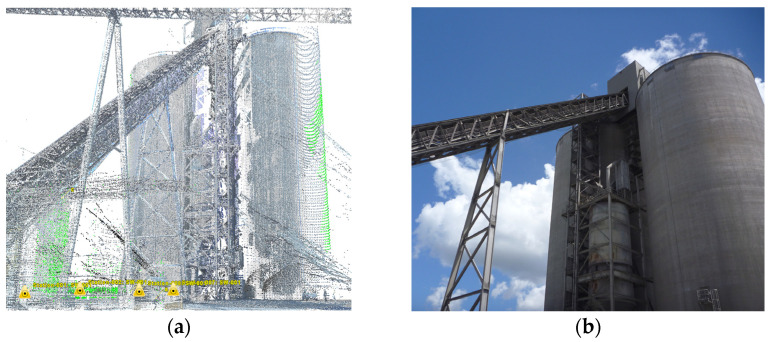
Points of clouds (**a**) obtained from 3D laser scan of industrial equipment (**b**).

The first step of the proposed method is to identify the geometry of the pressure equipment under test. The information obtained from the technical documentation is used for this purpose. These are primarily constructional drawings. An additional activity at this stage is verification and/or supplementary measurements on the tested facility. An additional, unique action introduced by the authors into the proposed method is the scanning of the device geometry. For this purpose, 3D laser techniques are used, which allow us to recreate the real construction geometry of the object in the virtual space of the computer-aided design system. The view of the point cloud obtained from the scan of an exemplary industrial installation is shown in Figure 2. The used 3D laser scanner uses 808 nm wavelength, which measures the distance using the interferometry technology. The head of the scanner rotates in horizontal and vertical axes. The combination of two-axis rotation results in measurement of a spherical coordinate system. The interferometry is realized by waveform digitizing technology (WFD), where a processor calculates distance based on the time difference between reflected laser signal and referent signal. This approach allows us to identify the real construction form of the object and validate the geometric model, which will be the basis for the further calculations. The accuracy of 3D laser scanning is relatively high and depends on the size of the tested object, scanning time, weather conditions (wind, rain, dust, scanned material reflectivity coefficient, etc.). For industrial facilities shown in the figure below, with a size of several meters up to several dozen meters, the accuracy ranges from a few mm to several cm. The scan of the object also allows for the detection of inconsistencies with the design, and also makes it possible to identify defects in the structure. These can be, for example, deformations, cracks and other defects manifested by a change in the geometry of the object in relation to the documentation/design version.

On the basis of the identified geometry, a geometric model of the examined object is built in a state corresponding to its real and current form. The geometric model is then used to build a discrete finite element model. In order to obtain high accuracy of the strength and durability calculations and to estimate the residual life, it is necessary to use detailed modeling of the tested objects. Depending on the required detail of the calculations (global or local analyzes), shell or volume models are used. In the case of pressure equipment, the proposed approach is to combine these two approaches and refine the models in the area of expected stress concentrations. These are usually areas of stubs, manholes, changes in direction and splitting of pipelines, etc. The use of shell models allows us to determine the effort of the walls of vessels, pipelines, supports, etc. with high accuracy. However, it is not possible to analyze in detail the influence of weld geometry in shell models. For this reason, solid modeling is used in regions that require such detail.

The next step is to identify the loads acting on the structure. This stage depends on the type of the tested object. The most complete source of information is the measurement of the operational loads. This can be done by one’s own research, after installing a measuring system that measures, e.g., pressure in the system, temperature, deformations and other parameters that enable their interpretation as a load. It has to be noted that such measurements provide information about the current load status. However, in the case of objects after many years of operation, they do not provide certainty whether these loads are representative of the entire period of operation. This has a very significant influence on the subsequent analyses of the residual life of the tested object. The use of data from monitoring systems for operating parameters of a given installation may be helpful here. If these systems archive measurement data, we can obtain very important information on the actual loads over a longer period of operation. This increases the accuracy of durability analyses using the hypothesis of fatigue failure accumulation. However, the history of loads from the entire service life of a given installation is rarely available. In such a situation, representative load blocks are assumed, defined on the basis of the available history and information on the operation technology. A quite typical situation in the operation of industrial pressure installations is the periodicity of their operation, resulting from the following factors:

- The nature of the technological process—periodicity is related to, e.g., the chemical process occurring during the operation of the installation, which affects changes in temperature, pressure, pulsation frequency, etc. The periodicity nature of such a process allows for the identification of representative load blocks and determination of their number, both in the past and planned future of exploitation.
- Installation outage cycles—in a typical approach to maintaining the technical condition of an installation, fixed periods of operation are used, followed by downtime and outage. In such a situation, it is possible to assume the cyclical nature of the load on the object related to the outage cycle.
- Cycles of technical condition tests—in the case of pressure equipment, the regulations/standards define the time intervals after which technical condition tests must be carried out, including appropriate tests confirming the safety of operation. These are, for example: pressure test, water test, NDT examinations and others. These tests are usually carried out during outage works. Loads occurring during such tests may also have an influence on the total durability of the installation, which, however, is usually neglected in residual life, durability analyzes. This topic will be discussed in more detail in the paper.

In the case of carrying out durability analyses along with the determination of the residual life, the identified representative load blocks allow us to determine the number of load cycles in individual ranges of amplitudes. A common method for determining the load spectrum is the rainflow counting method [17].

As is known, damage to pressure installations occurs at points of stress accumulation. In order to identify such places and determine the stresses distribution under operational loads, various calculation methods, both analytical and numerical, are used. The analytical methods have their limitations due to the lack of a strict solution in complex and statically indeterminate load conditions. Therefore, the most effective calculation methods are numerical methods, including the finite element method (FEM) [18]. Thanks to this, it is possible identify the state of stress with high precision, depending mainly on the detail modeling of the geometry, restraints and loads on the object. The already mentioned approach, combining shell and volume modeling, seems to be optimal for pressure equipment. In the case of pressure devices, it also allows for nonlinear analyses with consideration of the geometrical and material phenomena. In this type of facility, we deal with such phenomena (e.g., overstressing of the device during pressure tests).

Correctly identified loads and stress distribution conditions with the use of numerical methods in conjunction with data on load variability (rainflow) allows for fatigue analyses with the use of damage accumulation hypotheses. The most popular include:

- Palmgren–Miner rule. The damage function is described by the expression:

(1) $$D_{PM}=\sum_{i=1}^{q}\frac{n_{i}}{N_{i}}=1$$

where:

*D_PM_*—function of fatigue damage (failure) according to Palmgren–Miner hypothesis

*n_i_*—number of cycles with stress amplitude of σ_ai_,

*N_i_*—number of cycles to failure at stress amplitude of σ_ai_,

*q*—number of stress levels.

The main disadvantage of this theory is that the cumulative fatigue damage does not include stresses below fatigue limit. Therefore, the following hypotheses are more useful for cumulative fatigue damage:

- modified Palmgren–Miner rule—in this hypothesis the S-N curve of limited fatigue strength is extended at the same angle, defined as m—cotangent of the S-N curve slope angle. This modification of the hypothesis includes the stress-inducing loads below the fatigue limit.
- Haibach hypothesis—the S-N curve of limited fatigue strength is extended at an angle defined by exponent m’, which is related to exponent m in the following manner:
  m’ = 2m − 1
- Corten–Dolan hypothesis, described by the dependency:
  (2) $$D_{CD}=\sum_{i=1}^{q}{\left(\frac{n_{i}}{N_{i}}\right)}^{\rho (\sigma )}$$
  where *ρ(σ)* is the exponent which depends on the stress at individual levels in the time-trace, and which usually has a value of (0.8 ÷ 0.9) m.
- Serensen-Kogajev hypothesis, where the damage function has the form:
  (3) $$D_{S}=\sum_{i=1}^{q}\frac{n_{i}}{N_{i}}=a_{s}$$
  where:
  (4) $$a_{s}=\frac{\sigma_{a\max}\zeta -kZ_{g}}{\sigma_{a\max}-kZ_{g}}$$

*σ*_*a* max_—largest amplitude in the time-trace,

*k*—coefficient describing the sensitivity of the hypothesis by determining the smallest value of stresses that may cause fatigue damage (this value is usually assumed at 0.5),

*ζ*—duty cycle.

It is assumed that fatigue damage will occur when the sum *D* reaches the value of one. There are cases where this value is lower than one.

Depending on the results obtained from the conducted analyses, knowledge about the current technical condition of the facility and assessment of the safety of its further operation can be done. In many cases it appears that the fatigue life has been reached or that the residual life is unsatisfactory. In such a situation, it is possible to develop methods that extend the time of safe operation. For this purpose, the same simulation and computational tools are used in accordance with the method presented in Figure 1. Modeling various variants of modifications, reinforcements, object repairs and their fatigue verification allows for the development of an optimal modernization variant [19,20,21].

A detailed description of the proposed method with examples of application is presented later in the article.

## 3. Numerical Analyses of Pressure Vessels

**Figure 3:**
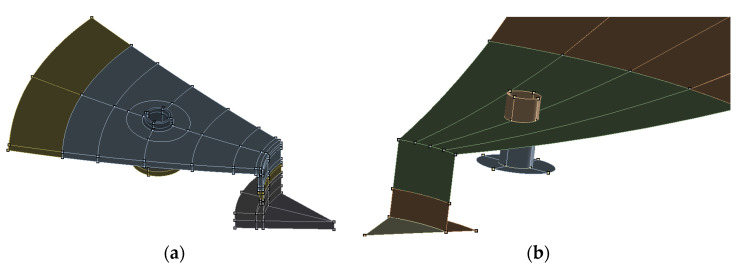
Geometrical model of part of the pressure vessel in area of stub and manhole modeled with solid modeling approach (**a**) and shell modeling approach (**b**).

Pressure equipment, mainly vessels, as opposed to typical load-bearing structures designed for high-cycle fatigue strength, are also subject to the phenomena of local plasticity. Moreover, in this type of object, there may also be a need to take into account geometric nonlinearities related to large deformations under load. Such situations especially occur during commissioning overload tests, as well as during periodic operational safety tests. With the complicated structure of the tank and the accompanying installation, as well as with complex load conditions, it may turn out that the scale of these phenomena is greater than assumed by the designer or operator of the equipment. In such a situation, a higher local stress effort is observed, e.g., in the areas of stub pipes, manholes, etc., which in an unfavorable situation may even lead to the occurrence of cyclic plastic deformations during the operation of the object. This condition is unacceptable as it leads to accelerated degradation resulting in often critical failures of the pressure equipment. In order to assess and prevent such phenomena, the proposed computational method using advanced numerical calculations can be used. The method enables a detailed analysis of the stress effort of the object for its various operating and test conditions. It allows the modeling of plasticity and hardening phenomena, also with cyclical load patterns. It requires detailed modeling of geometry, especially in stress concentration regions. For this purpose, shell and volume modeling are used. The view of an exemplary area of the pressure vessel modeled using the shell and volume approach is shown in Figure 3. More detail can be obtained in the volumetric model, but it requires much more computational resources, especially when conducting nonlinear material and geometric analysis. Therefore, this modeling method is usually used locally as a complement to the shell model.

In this particular case the first calculations were made for a tank modeled with the use of thin shell elements. On the basis of the obtained results, the occurrence of significant plastic deformations of a cyclical nature in the area of the lower manhole and lower spouts was found. Then, the obtained calculations were verified on a volumetric model taking into account a larger number of geometric details discretized by means of HEXA 8 elements.

**Figure 4:**
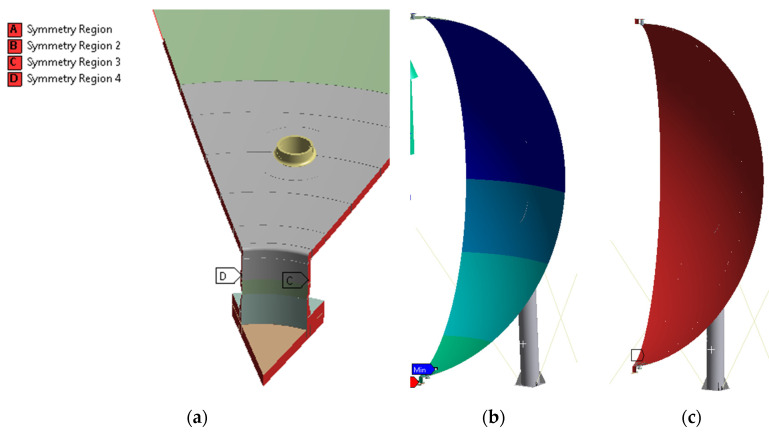
(**a**) Symmetry conditions applied to the 1/9 liquid nitrogen tank model, (**b**) hydrostatic pressure exerted by liquid nitrogen to the internal surfaces of the 1/9 liquid nitrogen tank model, (**c**) pressure from gases located above the liquid nitrogen mirror applied to the surface of the 1/9 liquid nitrogen tank model.

Figure 4a presents the symmetry conditions assumed for both the surface and outer side edges of both the volumetric and surface parts of 1/9 of the liquid nitrogen tank model. Under these symmetry conditions, appropriate volumetric finite elements (displacements perpendicular to symmetry planes) and shell elements (displacements perpendicular to symmetry planes and rotations along axes located in symmetry planes) were obtained. On the other hand, Figure 4b,c show the loads applied to 1/9 of the tank model, which are respectively liquid nitrogen hydrostatic pressure and pressure from gases located above the liquid nitrogen surface on the vessel walls.

**Figure 5:**
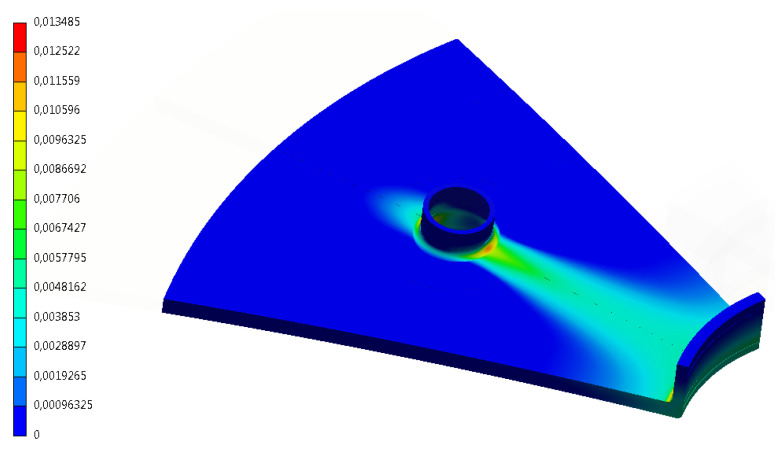
Plastic deformation zone in the pressure vessel, located between the stub pipe and the manhole [mm/mm].

Another phenomenon in pressure vessels that can be detected using numerical simulations is the mutual overlapping of geometric notches, resulting in a greater than theoretically determined local stress and strain effort. An example may be the location of several stub pipes or manholes on the surface of the tank in close proximity to each other. Considering each such element independently gives a standard and computationally correct result from the point of view of design requirements. However, the overall analyses show that this approach may lead to an underestimation of the stress and strain effort. Figure 5 shows an example of the influence of the manhole, located at the bottom of the pressure vessel, on the stress distribution around the connection stub located next to the manhole. There is an extensive zone of plasticization between the manhole and the stub pipe, which would not occur in the case of independent analyses of these two regions, without taking into account their mutual influence. Such situations can lead to premature depletion of the service life of the pressure vessel and its failure.

**Figure 6:**
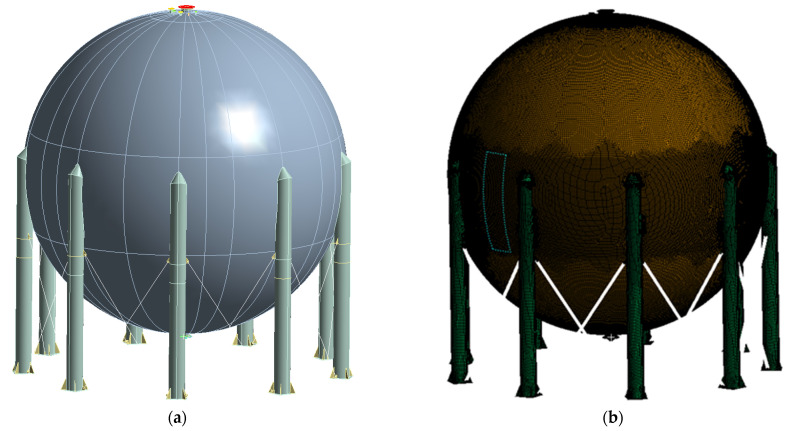
Shell model of the full geometry of a spherical pressure vessel (**a**), discrete model of the vessel (**b**).

An example of stress distribution identification for various operation and testing conditions in the spherical liquid nitrogen pressure vessel with the use of advance FEA simulations is presented below. The vessel of 2000 m^3^ capacity and 17 m diameter is made of steel plates. In the top and bottom areas there are multiply stubs and centrally located man holes present. For geometrical modeling purposes the vessel was made as a shell model for global calculations of the entire vessel. As a second step, local 3D modeling was introduced for more detailed simulations in highly stressed areas. A shell model was made by guiding the surfaces through the sheet metal middle thickness (Figure 6a). Geometric models have been discretized primarily using thin shell linear surface elements with thicknesses corresponding to the thicknesses of the actual structural plates of the vessel. In addition, rod elements (Link 180) which includes stress–stiffness terms in any analysis that includes large-deflection effects, were used to model cross-bars fixed within the supports of the vessel (Figure 6b).

For the definition of the finite element mesh for the considered models of liquid ammonia tank, the overall size of 150 mm was assumed. On the other hand, in areas critical in terms of potential stress concentrations, such as the places where the supports connect with the spherical surface and the trunks in the upper and lower part of the tank, the mesh was compacted to the size of 20 mm and 10 mm. Thanks to this approach, it was ensured that the results in places would be more accurate.

The discrete model includes models of various grades of structural steels, which have been used for the structural components of the vessel, such as steel plates for the vessel sphere, stubs, man holes, vertical support columns.

Numerical calculations of the vessel structure were performed using the finite element method in the Ansys Workbench environment. During this analysis, linear elastic material models of individual steel grades were taken into account (no material nonlinearity), while maintaining geometric nonlinearity resulting from potentially large vessel displacements under the load.

The following design cases were considered during simulations:

- **Case 1**—loading: gravity, gas pressure inside the vessel equal to 1.0 MPa (case with unit load of internal pressure for further fatigue calculations),
- **Case 2**—loading: gravity, maximum operational gas pressure inside the vessel equal to 1.6 MPa (the case to assess the effect of maximum operational pressure in the vessel),
- **Case 3**—loading: gravity, hydrostatic pressure from liquid ammonia when the vessel is filled to 21% of its volume, which is minimum design liquid level in the vessel (the case to assess the effect of filling the vessel with liquid ammonia),
- **Case 4**—loading: gravity, hydrostatic pressure from liquid ammonia with the vessel filling 69% of its volume, which is the maximum design liquid level in the vessel (case to assess the effect of filling the vessel with liquid ammonia,
- **Case 5**—acting gravity, hydrostatic pressure from liquid ammonia, when the vessel is filled to 69% of its volume, gas pressure inside the vessel is 1.6 MPa.

Case 1 is defined for further fatigue calculations. Cases 2–4 are defined to assess the influence of operational loads (liquid level and gas pressure) on the stress distribution. Case 5 refers to maximum operational loads condition.

The defined load case purposes are to determine their individual impact on the actual state of stress in the structure of tanks of liquid nitrogen. This in turn allowed us to assess which kind of stress mainly decides about the loss of durability of these structures.

**Figure 7:**
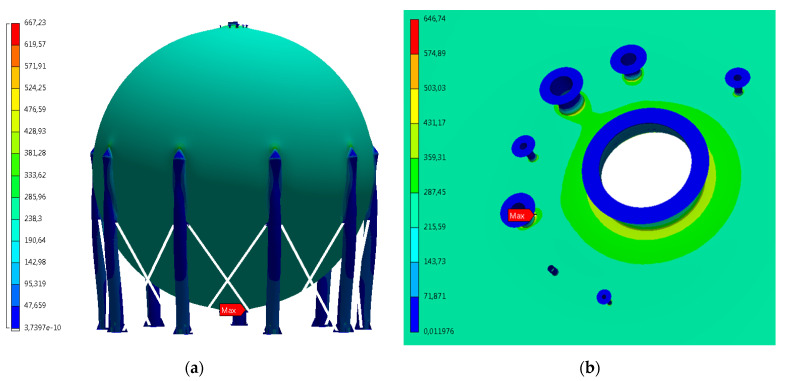
Equivalent von Mises stress contours [MPa] of the structure of the vessel for load case 5. View of the entire vessel (**a**), maximum stresses in the area of the stub pipe in the lower part of the vessel (**b**).

Examples of calculation results for load case 5, in the form of equivalent stress contours according to the Huber–Mises hypothesis are shown in Figure 7.

Analyzing the obtained calculation results, it was noticed that the most stressed regions of the vessel are the places where the vessel shell connects with the stub pipes in the lower and upper part of the vessel, centrally located manholes and support columns. Moreover, for design case 5 (during the maximum working load of the vessels), obtained stress level indicates presence of local plastic zones (stress level above yield point).

**Figure 8:**
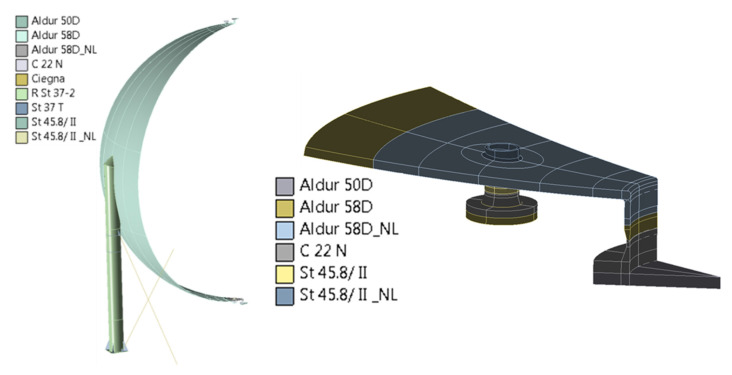
Distribution of materials with nonlinear characteristics on the vessel structure model in the lower manhole and nearby stubs (the designation NL next to the name of the steel grade means a nonlinear material model).

Due to the occurrence of exceedance of the yield point under operational loads, a material nonlinear analysis of the vessel was carried out with consideration of 3D modeling approach in the highly stressed areas (bottom stubs and manhole areas). For this purpose and with consideration of the complexity of these calculations, the 1/9 model of the vessel geometry was used for this simulation. Material nonlinearity of selected steel elements of the vessel was assumed in the area of the shell connection with the support and in the area of the lower manhole and nearby stubs. The exact distribution of materials with nonlinear characteristics considered in the model is presented in Figure 8.

For the simulation of the overload of the reservoir during the water test, taking into account plastic deformations, elastoplastic models of materials were defined, taking into account the effect of kinematic strengthening. As a result, the data including deformations and the corresponding stresses were entered into the calculation program as actual values, which are summarized in Table 1.

**Figure 9:**
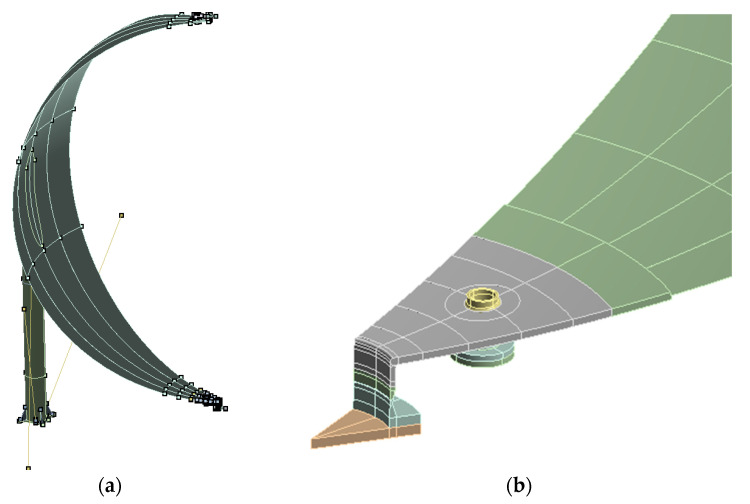
Geometric model of spherical tanks used in the stress analysis (**a**) solid surface model—general view (**b**) area detailed by means of a volumetric model.

While the axisymmetric model enabled us to run the simulation with 1/9 geometry, the simulation time was relatively acceptable while all the material model was nonlinear. It was also beneficial due to the fact that part of the model was modeled with use of the solid finite elements. Figure 9 depicts the generic view of the 1/9 of the geometry and the close-up to the solid to shell transition area.

**Figure 10:**
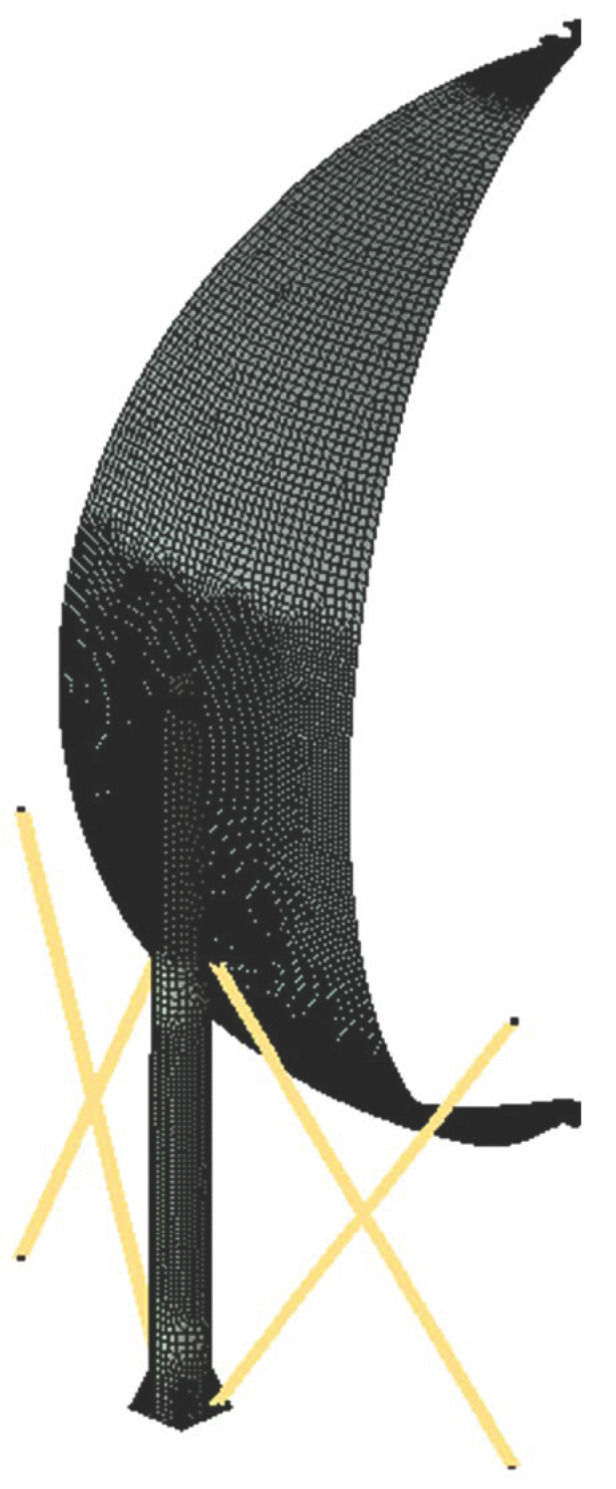
Discrete model—the general view.

**Figure 11:**
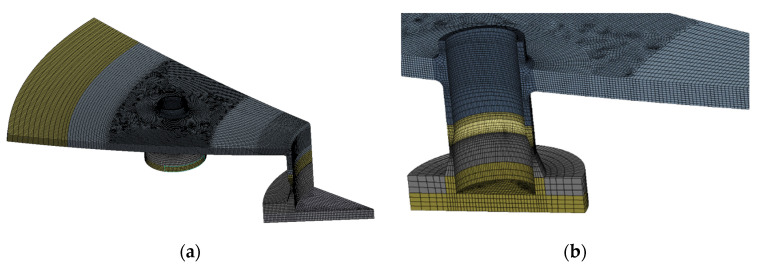
Solid element (**a**) general view, (**b**) detailed view of the stub.

The geometry prepared in this way was discretized using HEXA 8 volumetric elements with 3 degrees of freedom in the node (detailed area of the tank model), thin shells with 6 degrees of freedom in the node and thicknesses corresponding to the thickness of the sheets (the remainder of the model with a lower level of detail) and rod-shaped tensile stiffening the columns support the structure. The model developed in this way consisted of 302 732 terminated elements and 339 145 nodes, which gave the number of degrees of freedom equal to 1 085 004. The discrete model is presented in Figure 10 (rod, shell and solid elements) and the detailed view of the solid discretization is presented in Figure 11.

**Figure 12:**
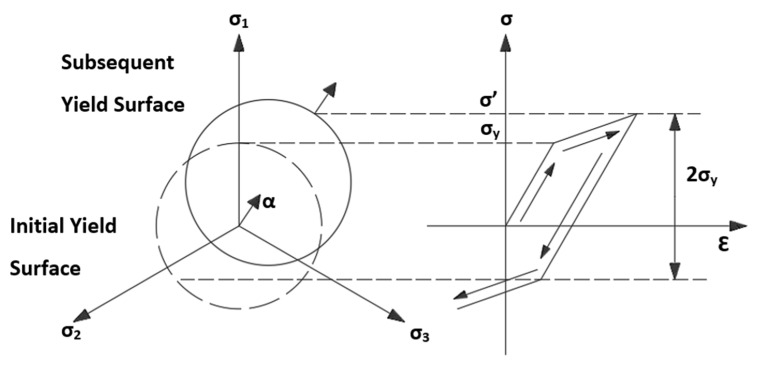
Distribution mechanism of kinematic strengthening.

Due to the prevailing kinematic character of the hardening of the construction material, this hardening model was used in numerical calculations. It consists in the displacement of the plastic surface in the direction of the load, with the simultaneous lack of change in both its size and shape Figure 12.

The yield criterion for kinematic hardening can be described by the following equation:

(5) $$F=\sqrt{\frac{3}{2}\left(s-\alpha \right):\left(s-\alpha \right)}-\sigma_{y}=0$$

where *s* is the deviatory stress, *σ_y_* is the uniaxial yield stress, *α* is the back stress (location of the center of the yield surface).

The back stress is described by the equation below:

(6) $$\Delta \alpha =\frac{2}{3}C\Delta \varepsilon_{pl}$$

**Figure 13:**
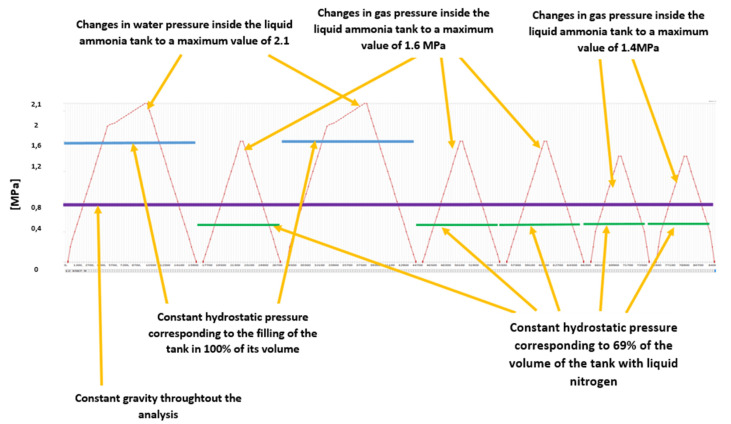
The load scheme adopted to carry out nonlinear numerical calculations taking into account the overloading of tanks with the use of a water tests and then standard operational conditions.

The nonlinear analysis carried out for 1/9 of the vessel part consisted of 259 steps. All testing and operational conditions were considered within these steps. Analyses included water and pressure tests of the vessel during which it is filled with water in 100% of its volume. Between these two tests, one operating condition is simulating (with maximum operating liquid volume of 69% and 1.6 MPa of gas pressure) This is to control the overstress of the vessel, which allows to reduce its stresses as a result of loads appearing during normal operation. The following two water tests the load scheme included 4 operational load cycles (2 cycles with 16 MPa, gas pressure and 2 with reduced pressure of 1.4 MPa). The entire load scheme with detailed description is presented in Figure 13.

**Figure 14:**
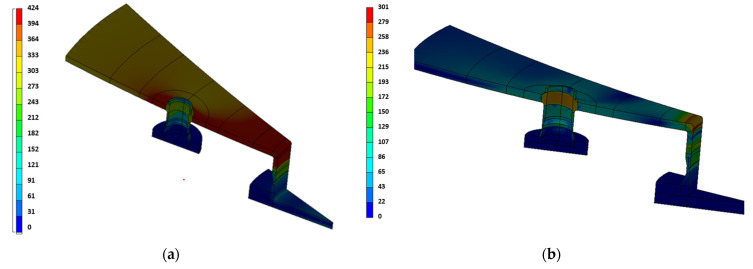
Contours of von Mises stresses within the lower manhole of the vessel for the time t = 9150 s, for which they reach the maximum value (**a**), for the time t = 83,910 s—corresponding to the condition of the unloaded tank (gravity only) residual stresses (**b**).

Selected results of numerical calculations for contour’s specific forms of von Mises stresses are shown in the figures below (Figure 14).

For two of areas with significant plastic strains observed, more detailed analyses are presented below:

- P1v1—stub to vessel shell connection area,
- P4v2—bottom manhole to vessel shell connection area.

**Figure 15:**
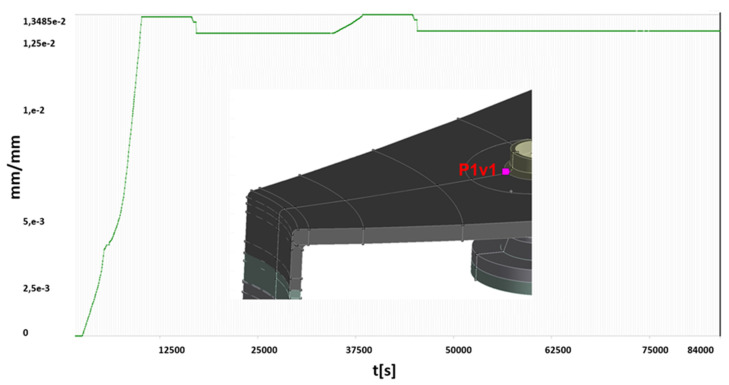
Generalized plastic strain for the entire loading scheme—point P1v1.

**Figure 16:**
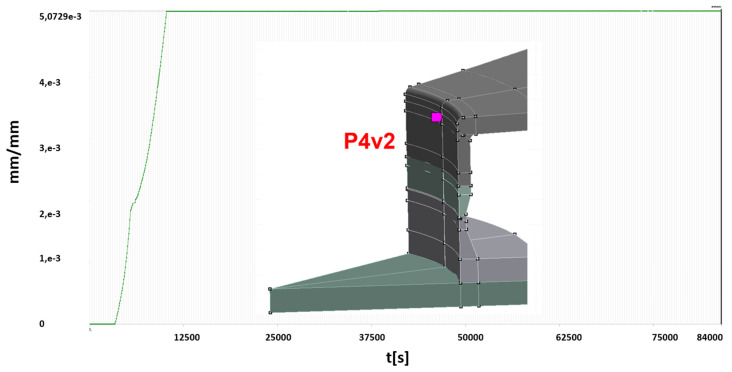
Generalized plastic strain for the entire loading scheme—point P4v2.

In order to conduct an in-depth analysis of the formation and development of plastic strains in the abovementioned areas the courses of changes of the plastic strains during the entire simulation (loading cycles shown in Figure 13) were determined. Time traces of changes of the generalized plastic strain for the above points are presented (Figure 15 and Figure 16).

Based on the obtained calculation results, the following conclusions can be given:

- in the area of the lower stub to shell connection (point P1v1), cyclic plastic deformations are observed for test conditions. This means that each tests causes plastic deformation of this area, which is not expected.
- higher than expected plastic deformations are caused by location of the stub near the manhole, which changes stress distribution in this area significantly.
- in the remaining areas of the vessel, no development of plastic zones was found after overstressing the vessel (the first water test).

As shown in the presented example only detailed, nonlinear simulations can identify areas where potential failure may occur. Cyclic plastic deformation leads to cracks in few cycles. With such information from FEA simulation, it is possible to avoid or minimize such cyclic plastic deformation conditions.

Another very important topic related to safety of operation of pressure vessel is residual life estimation. This subject is discussed in the next chapter.

## 4. Fatigue Calculations of Pressure Equipment

Many years of operation in variable load conditions requires an assessment of the residual life, taking into account the history of loads. In such a case, the best basis for calculations is a detailed identification of the stress/strain distribution, with the use of shell and/or volume models (discussed in Section 3 of the paper) and a representative history of loads. With such data available, it is possible to estimate the service life using the fatigue accumulation hypotheses described in the paper. An example of such a calculation for a pressure vessel is shown below.

### 4.1. Loads Identification for Fatigue Calculations

In the presented example it was possible to obtain history of loads of the circular pressure vessel (presented in Section 3) from the last 3 years of operation. These data consist of:

- percentage change of the liquid level in the vessel,
- the vessel internal pressure,
- number of emptying of the vessel per year,
- number and conditions of tests (water, pressure tests).

**Figure 17:**
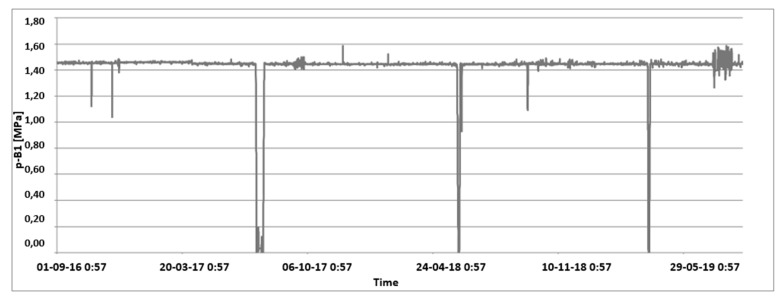
Annual internal pressure in the vessel change.

Example of time loads in a form of internal pressure in the vessel is presented in Figure 17.

### 4.2. Fatigue Calculations

**Figure 18:**
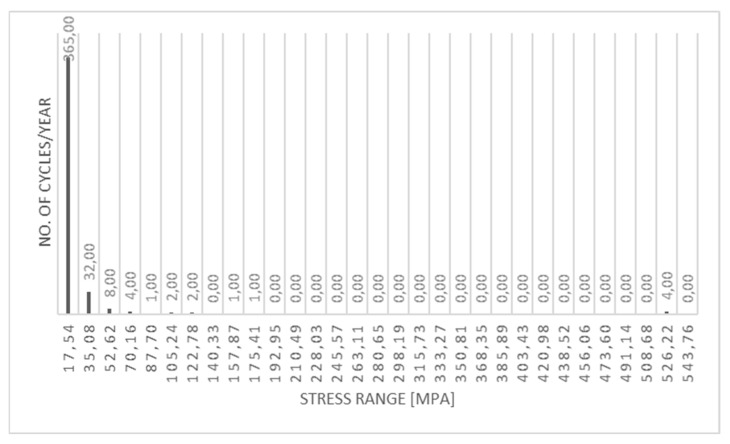
Stress range rainflow chart, point P1v1, [MPa].

In the fatigue calculation, cumulative damage approach described in Section 2 was used. For stress identification in the vessel structure, FEA model and simulation results described in the Section 3 were used. As a result, stress range variations for separate loads were obtained. As a next step, rainflow counting was applied to enable calculations of fatigue damage in each location of the pressure vessel. Figure 18 presents example of the stress range rainflow chart is presented for point P1v1. In these calculations fatigue properties of welded connections as well as out of welds areas were considered based on EN 1993-1-9:2005 standard (Eurocode 3: Design of steel structures—Part 1–9: Fatigue).

As a result, cumulative damage, which consists of damages from all loads, was calculated and residual fatigue life obtained as well. As expected, the most critical location, where the lowest residual fatigue life was calculated, is the area of the lower stub to shell connection (point P1v1), which equals:

N_fat_ = 190 year

Based on these calculations, it was also possible to estimate the impact of periodic water and pressure tests of the vessel. It was calculated that each such test reduces fatigue life for 17 years of operation.

## 5. Discussion and Conclusions

The presented method makes it possible to detect phenomena that are difficult to take into account or have not been included in the research on this type of object conducted so far. It is particularly important to be able to assess the mutual influence of irregularities in the construction of a pressure vessel (e.g., stubs, manholes). The presented calculations of the pressure vessel show that this influence is significant and may lead to underestimating the strength and durability of the pressure vessels in the design process. Thanks to detailed numerical models and advanced nonlinear simulations, it was possible to determine the weakest area of the vessel, which has not been identified previously. The stub located next to the manhole turned out to be the most critical area of the vessel with the lowest residual life calculated of 190 years of operation in typical conditions. In the calculations, it was assumed that loads (amplitude, number of cycles) will not change in the future. Moreover, it was determined that periodic pressure tests required by regulations reduce this residual life for 17 years of operation due to periodical plastic de-formation present during such overload tests. Based on obtained results, the decision was made to change pressure and water testing methods and parameters to acoustic emission method, to avoid the primary method negative impact on durability.

Detailed knowledge about the condition of the pressure vessel allows for its safe operation. It also allows us to make justified and correct decisions about operating life. Precise identification of weak points and the possibility of their modification before damage occurs, allows us to extend this time of safe operation, and thus make better use of the installation. The use of detailed numerical models (shell and solid modeling), taking into account the actual geometry of the tested object (3D scanning) and the use of representative load traces (operation history, measurements), ensure very good precision of the entire investigation.

An additional advantage of the presented approach is the possibility of assessing and, if necessary, correcting the scope and/or the method of operating tests of the pressure vessel, in the event of their negative impact on durability. In such a case, the test conditions may be modified or the test method may be changed (e.g., the acoustic emission method may be used instead of the pressure test).

The presented example of pressure vessel calculations shows the abovementioned phenomena and describes the details of the application of the developed method.

The presented method for the assessment of the technical condition of pressure equipment may be further developed through the use of new research, measurement and calculation techniques. It depends on the research object, phenomena occurring in it (e.g., thermal phenomena), as well as expectations as to the detail of the obtained results of the investigations. It can also be used for development of methods that increase durability and extend the time of safe operation of pressure equipment. Additionally, the presented method stands as a great basis for the development of the technical condition monitoring systems of pressure equipment.

## Figures and Tables

**Table 1:** Values of deformations and actual stresses of individual construction materials of tanks introduced into elastoplastic models taking into account the phenomenon of kinematic strengthening.

1	Type of Steel	Yield Point (Actual Values) Re [MPa]	Endurance Limit (Actual Values) Rm [MPa]	Plastic Deformation at the Moment of Sample Breaking (Actual Values) [mm/mm]	Relative Extension [%]
1	ALDUR 58D	402	752	0.230	26.3
2	ALDUR 58G	402	681	0.196	22.0
3	ALDUR 50D	353	616	0.255	29.4
4	R St 37-2	255	432	0.188	21.0
5	ST 37	230	370	0.205	23.0
6	St 45.8/II	254	525	0.188	21.0
7	C22	245	518	0.170	19.0
8	Alfrog44	264	527	0.220	25.0

## Data Availability

Not applicable.
